# Deficiency of superoxide dismutase promotes cerebral vascular hypertrophy and vascular dysfunction in hyperhomocysteinemia

**DOI:** 10.1371/journal.pone.0175732

**Published:** 2017-04-17

**Authors:** Sanjana Dayal, Gary L. Baumbach, Erland Arning, Teodoro Bottiglieri, Frank M. Faraci, Steven R. Lentz

**Affiliations:** 1 Department of Internal Medicine, University of Iowa Carver College of Medicine, Iowa City, Iowa, United States of America; 2 Department of Pathology, University of Iowa Carver College of Medicine, Iowa City, Iowa, United States of America; 3 Baylor Institute of Metabolic Disease, Dallas, Texas, United States of America; 4 Department of Pharmacology, University of Iowa Carver College of Medicine, Iowa City, Iowa, United States of America; University of Louisville, UNITED STATES

## Abstract

There is an emerging consensus that hyperhomocysteinemia is an independent risk factor for cerebral vascular disease and that homocysteine-lowering therapy protects from ischemic stroke. However, the mechanisms by which hyperhomocysteinemia produces abnormalities of cerebral vascular structure and function remain largely undefined. Our objective in this study was to define the mechanistic role of superoxide in hyperhomocysteinemia-induced cerebral vascular dysfunction and hypertrophy. Unlike previous studies, our experimental design included a genetic approach to alter superoxide levels by using superoxide dismutase 1 (SOD1)-deficient mice fed a high methionine/low folate diet to produce hyperhomocysteinemia. In wild-type mice, the hyperhomocysteinemic diet caused elevated superoxide levels and impaired responses to endothelium-dependent vasodilators in cerebral arterioles, and SOD1 deficiency compounded the severity of these effects. The cross-sectional area of the pial arteriolar wall was markedly increased in mice with SOD1 deficiency, and the hyperhomocysteinemic diet sensitized SOD1-deficient mice to this hypertrophic effect. Analysis of individual components of the vascular wall demonstrated a significant increase in the content of smooth muscle and elastin. We conclude that superoxide is a key driver of both cerebral vascular hypertrophy and vasomotor dysfunction in this model of dietary hyperhomocysteinemia. These findings provide insight into the mechanisms by which hyperhomocysteinemia promotes cerebral vascular disease and ischemic stroke.

## Introduction

Hyperhomocysteinemia, or elevation of plasma total homocysteine (tHcy)[1], is associated with excess risk of both cardiovascular and cerebral vascular events [2]. Prospective association studies have demonstrated that hyperhomocysteinemia confers only a modestly increased relative risk for coronary events but has a larger impact on the relative risk for stroke [3]. Consistent with these findings, homocysteine-lowering therapy does not prevent secondary cardiovascular events [4–9] but does decrease incident stroke in both primary and secondary settings [7,8,10–13]. The landmark China Stroke Primary Prevention Trial found that homocysteine-lowering therapy with folic acid significantly reduced the risk of first stroke in hypertensive adults treated with the anti-hypertensive medication enalapril [14]. These clinical observations suggest a causal role for elevated tHcy in cerebral vascular pathophysiology.

Several distinct abnormalities of cerebral vascular structure and function have been observed in murine models of hyperhomocysteinemia. These include endothelial vasomotor dysfunction, vascular hypertrophy and remodeling, and altered vascular permeability [15–18]. A prominent molecular feature of the aberrant cerebral vascular phenotype of hyperhomocysteinemia is vascular oxidative stress, with elevated levels of reactive oxygen species (ROS) [19]. For example, levels of ROS such as superoxide and hydrogen peroxide are elevated in cerebral blood vessels of mice with diet-induced hyperhomocysteinemia [16,17], and nonspecific pharmacological antioxidants can improve endothelial function [19]. However, this prior work did not define the specific mechanistic role of superoxide in causing the alterations of cerebral vascular structure and function that occur in hyperhomocysteinemia.

To gain further insight into the redox regulation of cerebral vascular structure and function during hyperhomocysteinemia, we took a genetic approach. Using mice with a targeted deletion of the murine *Sod1* gene, we examined the effects of deficiency of superoxide dismutase 1 (SOD1; the most abundant cellular isoform of superoxide dismutase in vascular tissues [20]) on vasomotor function and vascular structure in cerebral blood vessels of mice with diet-induced hyperhomocysteinemia. Hyperhomocysteinemia is a risk factor for small vessel disease in brain, a key contributor to stroke and cognitive deficits [21]. For this reason, we focused our efforts on cerebral microvascular changes in these experiments. Our findings implicate superoxide and superoxide-derived ROS as key redox mediators of abnormal cerebral vascular structure and function in hyperhomocysteinemia.

## Materials and methods

### Materials

Control mouse chow (LM485) and high methionine/low folate (HM/LF) diet (TD00205) were purchased from Harlan Teklad (Madison, WI). EDTA (AM9260G) was purchased from Thermo Fisher Scientific (Waltham, MA). Polyethylene glycol-superoxide dismutase (PEG-SOD, S9549), acetylcholine (A-6625), nitroprusside (S-0501), papaverine (P-3510), CaCl dihydrate (223506), MgCl_2_ hexahydrite (M9272), and D-glucose (G5767) were purchased from Sigma (St Louis, MO). Dihydroethidium (DHE; D11347) was purchased from Invitrogen (Carlsbad, CA). Sodium pentobarbital (NDC 76478-501-50) was purchased from Oak Pharmaceuticals, Inc. (Lake Forest, IL). NaCl (S23025), NaHCO_3_ (S25060) and KCl (P41025) were purchased from Research Products International Corp. (Mount Prospect, IL). Artificial CSF was prepared with the following ionic composition [mmol/L]: NaCl 132, KCl 2.95, CaCl_2_ 1.71, MgCl_2_ 0.65, NaHCO_3_ 24.6, D-glucose 3.69. Glutaraldehyde (111-30-8) was purchased from Electron Microscopy Sciences (Hatfield, PA).

### Mice and experimental protocol

Breeding pairs of heterozygous *Sod1*-deficient (SOD1tm1Leb) mice on a mixed B6/129S background were obtained from The Jackson Laboratory (Bar Harbor, Maine). Littermate offspring were genotyped for the targeted and wild type *Sod1* alleles in the University of Iowa Genome Editing Facility as described previously [22]. Starting at 4 weeks of age, wild-type (*Sod1*+/+), heterozygous (*Sod1*+/-), and homozygous (*Sod1*-/-) littermate mice were fed either a control diet containing 6.7 mg/Kg folic acid and 4.0 g/Kg L-methionine or a HM/LF diet containing 0.2 mg/Kg folic acid and 8.2 g/Kg of L-methionine [23]. Mice were fed the diets for 5–10 months before vascular studies were performed (S1 Fig). All animal protocols were approved by the University of Iowa Animal Care and Use Committee.

### Plasma tHcy

Blood was collected from mice anesthetized with sodium pentobarbital (75–90 mg/kg IV) by cardiac puncture into EDTA (final concentration 5 mmol/L), and plasma was collected after centrifugation. Plasma tHcy, the total concentration of homocysteine after quantitative reductive cleavage of all disulfide bonds [1], was measured by high-performance liquid chromatography (HPLC) and SBDF (ammonium 7-fluorobenzo-2-oxa-1,3-diazole-4-sulphonate) fluorescence detection [24].

### Detection of vascular superoxide

The oxidative fluorescent dye, DHE, was used to detect superoxide in frozen sections of the common carotid artery by laser scanning confocal microscopy as described previously [25]. Where indicated, sections were preincubated for 30 minutes with 250 U/mL PEG-SOD before incubation with DHE. Fluorescent images were analyzed with Scion Image software (Scion, Frederick, MD). Data are reported as the percentage of surface area of carotid sections within the upper 20% of fluorescence intensity. Average fluorescence was calculated relative to *Sod1*+/+ mice fed the control diet, which was set at 1.

### Vasodilation of cerebral arterioles

Dilation of cerebral arterioles was measured as described previously [16]. Briefly, mice were anesthetized with sodium pentobarbital (75–90 mg/kg IV that was supplemented regularly at approximately 20 mg/kg/hour) and ventilated mechanically. A cranial window was created over the left parietal cortex and a segment of a randomly selected pial arteriole (~30 μm in diameter) was exposed. The diameter of the cerebral arteriole was measured under control conditions and during superfusion with acetylcholine (10^−6^ and 10^−5^ M), nitroprusside (10^−8^ and 10^−7^ M), or papaverine (10^−6^ and 10^−5^ M) using a video microscope coupled to an image-shearing device. Acetylcholine induces dilation of cerebral arterioles by stimulating the endothelium to generate NO, which then activates guanylate cyclase in vascular muscle, leading to vasodilation [26]. Nitroprusside is an NO donor that bypasses the need for endothelium by directly releasing NO that induces dilation by activating guanylate cyclase in vascular muscle [26]. Papaverine acts directly on vascular muscle through a mechanism that is independent of both endothelium and NO [27,28]. Data were quantitated as the % change (Δ) in vessel diameter under control conditions vs. after superfusion with vasodilators.

### Cerebral arteriolar pressure, diameter and mechanics

Systemic arterial pressure in conscious mice was measured as described previously [15]. Mice were weighed and anesthetized with sodium pentobarbital (5 mg/100 g IV), intubated, and mechanically ventilated with room air and supplemental O_2_. Additional anesthesia (sodium pentobarbital 1.7 mg/100 g, IV) was administered when pressure to a paw evoked a change in blood pressure or heart rate. Both femoral arteries were catheterized to record systemic arterial pressure, obtain blood samples for measurement of arterial blood gases, and withdraw blood to induce hypotension for study of vascular mechanics.

Pressure and diameter in first-order arterioles on the cerebrum in a cranial window preparation was measured as described previously [15]. Cerebral arteriolar systolic, diastolic, mean and pulse pressures were measured continuously with a micropipette connected to a servo-null pressure measuring device [15,29]. Arterioles were monitored through a microscope connected to a closed-circuit video system. Arteriolar diameters were measured from digitalized images using NIH Image J analysis software (Bethesda, MD). Measurements of arterioles were obtained at baseline, followed by complete deactivation of smooth muscle layer by suffusion of cerebral vessels with artificial CSF containing 67 mmol/L EDTA. Pressure diameter relationships were obtained in deactivated cerebral arterioles. Maximally dilated arterioles were fixed at physiological pressure *in vivo* by superfusion of vessels with 2.25% glutaraldehyde while maintaining cerebral arteriolar pressure at baseline levels. Finally, blood was drawn from the femoral artery for measurements of plasma tHcy and mice were euthanized by injection of potassium chloride. The arteriolar segment used for pressure-diameter measurements was harvested, processed for electron microscopy and embedded in Spurr’s low viscosity resin while cross-sectional orientation was maintained. Circumferential stress, circumferential strain and tangential elastic modulus were calculated as described previously [15].

### Determination of cerebral arteriolar wall cross-sectional area and composition

The cross-sectional area of the vessel wall was measured histologically from 1 μm sections using a light microscope interfaced with the image analyzing system described above. Luminal and total cross sectional areas of the arterioles were measured by tracing the inner and outer edges of the vessel wall. The cross-sectional area of the arteriolar wall was calculated by subtracting luminal cross-sectional area from total cross-sectional area. The cross-sectional area and volume density of different components of vessel wall (smooth muscle, elastin, collagen, basement membrane, and endothelium) were quantitated from electron micrographs of vessel wall as described previously [15].

### Statistical analysis

Two-way analysis of variance (ANOVA), followed by Tukey’s post-hoc test for pairwise comparisons, was used to analyze the effects of *Sod1* genotype and diet on plasma tHcy, responses to vasodilators in cerebral arterioles, and measurements of arteriolar wall thickness and composition. Correlation coefficients were calculated using the Pearson product moment correlation. A value of P < 0.05 was used to define statistical significance. Values are reported as mean ± SEM.

## Results

### Effects of diet and *Sod1* genotype on plasma tHcy and vascular superoxide

**Fig 1:**
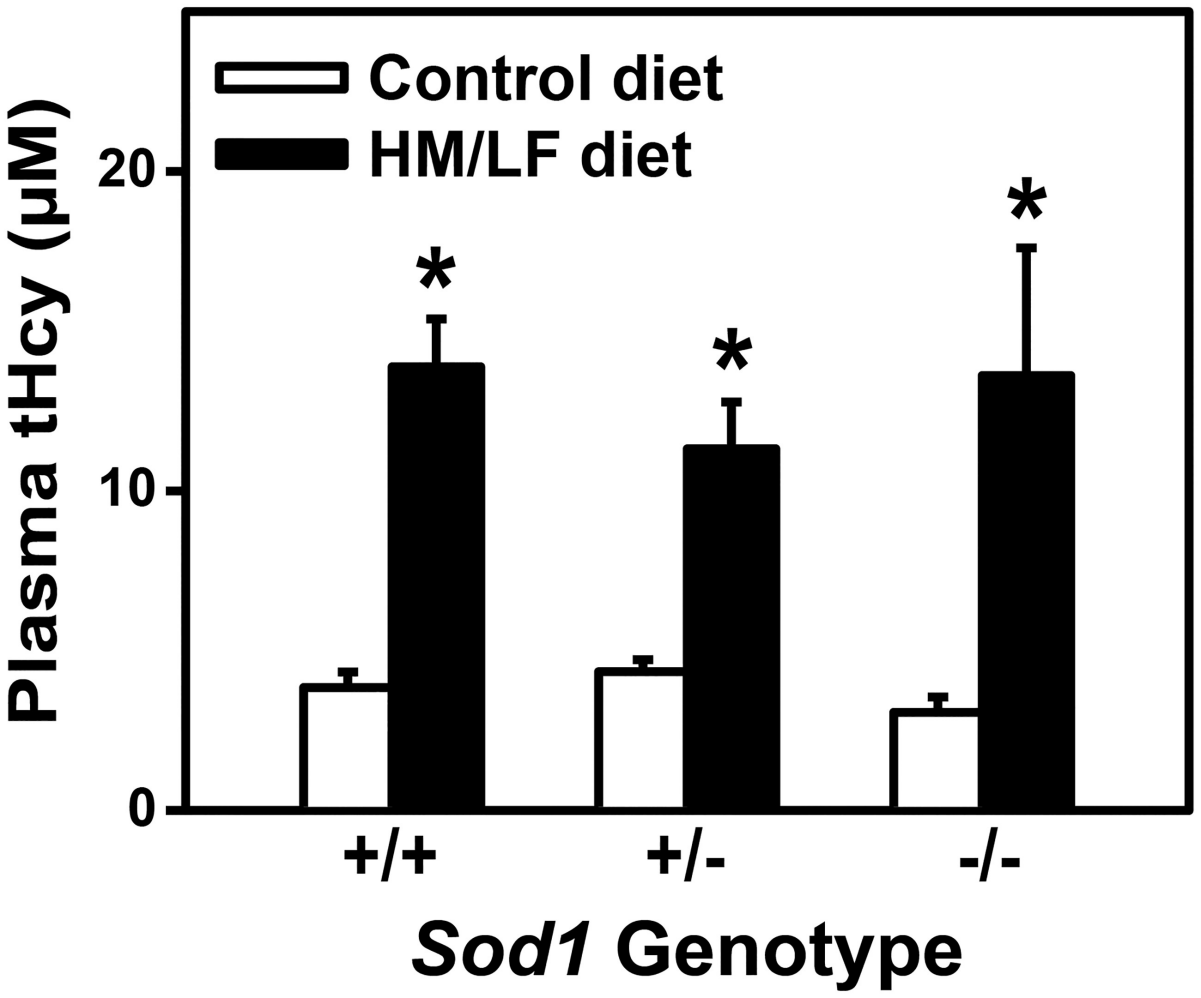
Plasma total homocysteine levels are elevated with the HM/LF diet. Plasma levels of tHcy were measured in *Sod1+/+*, *Sod1+/-*, and *Sod1-/-* mice on either control or HM/LF diet (n = 11–15 mice per group). Values are mean ± SEM. *P < 0.05 compared to control diet.

Wild type (*Sod1+/+*), heterozygous (*Sod1+/-*), and homozygous (*Sod1-/-*) littermate mice were fed either a control diet or a HM/LF diet to produce hyperhomocysteinemia. Plasma levels of tHcy were elevated in *Sod1*+/+, *Sod1*+/-, and *Sod1-*/- mice fed the HM/LF diet compared with mice fed the control diet (P < 0.01, Fig 1). Mean plasma tHcy levels were between 3.1 and 4.3 μmol/L in mice fed the control diet, and between 11.3 and 13.9 μmol/L in mice fed the HM/LF diet. This degree of elevation of plasma tHcy is similar to that observed in humans with mild or moderate hyperhomocysteinemia [3]. Plasma tHcy levels did not differ significantly between *Sod1*+/+, *Sod1*+/-, and *Sod1-*/- mice, indicating that the hyperhomocysteinemic response to the HM/LF diet was not altered by deficiency of SOD1.

**Fig 2:**
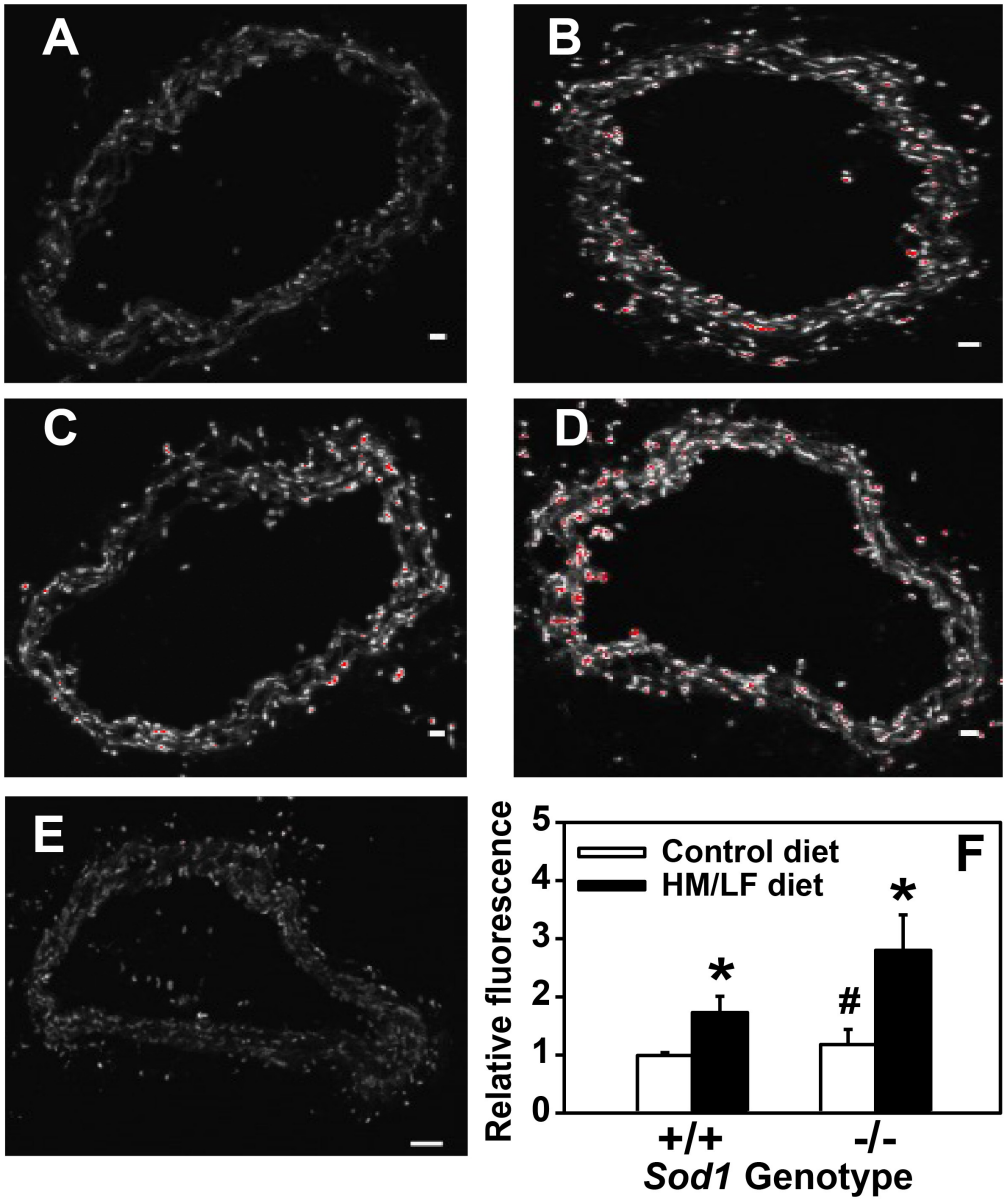
Vascular superoxide is increased with the HM/LF diet and SOD deficiency. Representative confocal fluorescent images of DHE-stained carotid artery sections as a measure of superoxide from (A) *Sod1+/+* mice fed control diet, (B) *Sod1+/+* mice fed HM/LF diet, (C) *Sod1-/-* mice fed control diet, and (D) *Sod1-/-* mice fed HM/LF diet. (E) Carotid artery section from *Sod1-/-* mice fed HM/ LF diet preincubated with PEG-SOD prior to DHE staining. Scale bar = 50 μm. (F) DHE fluorescence relative to *Sod1*+/+ mice fed the control diet (n = 5–9 mice per group). Values are mean ± SEM. *P<0.01 compared to control diet; # P < 0.05 compared to *Sod1+/+* on the same diet.

DHE fluorescence was measured *in situ* as an indicator of vascular superoxide levels. As expected [22,30], we detected increased DHE fluorescence in response to either the HM/LF diet (P < 0.01 vs. control diet, Fig 2) or deficiency of SOD1 in *Sod1*-/- mice (P < 0.05 vs. *Sod1+/+* mice, Fig 2). Deficiency of SOD1 in the context of the HM/LF diet resulted in a further increase in carotid artery DHE fluorescence as compared with *Sod1-/-* mice fed the control diet (P <0.05, Fig 2). Preincubation of the arterial sections with PEG-SOD diminished DHE fluorescence by >90% in *Sod1-/-* mice fed the HM/LF diet (Fig 2E), which confirms that the increased vascular DHE fluorescence observed in these mice is superoxide-dependent.

### Effects of SOD1 deficiency and hyperhomocysteinemic diet on vasomotor responses

**Fig 3:**
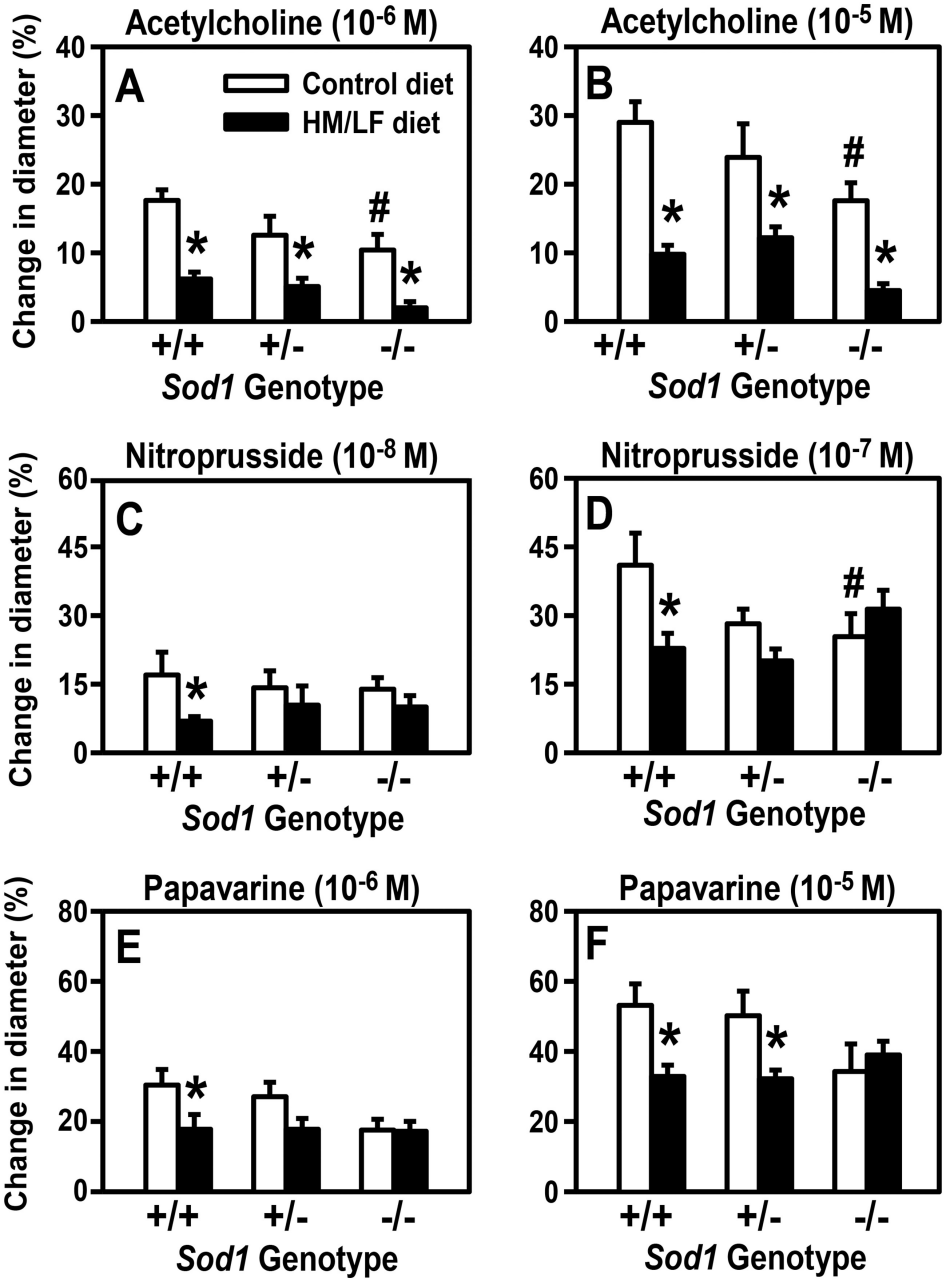
SOD1 deficiency and the HM/LF diet produce an additive increase in endothelial dysfunction and induce a non-additive decrease in vasodilator responses to nitroprusside and papaverine. Dilatation of cerebral arterioles to acetylcholine (10^−6^ M [A] or 10^−5^ M [B]), nitroprusside (10^−8^ M [C] or 10^−7^ M [D]), and papaverine (10^−6^ M [E] or 10^−5^ M [F] was measured in *Sod1+/+*, *Sod1+/-*, and *Sod1-/-* mice on either control or HM/LF diet (n = 7–10 mice per group). Values are mean ± SEM. *P<0.01 compared to control diet; # P < 0.05 compared to *Sod1+/+* on the same diet.

We next determined the combined influence of *Sod1* genotype and the HM/LF diet on endothelium-dependent vasodilator responses in cerebral arterioles using acetylcholine, which stimulates release of endothelial nitric oxide (NO). All groups of mice exhibited concentration-dependent vasodilator responses to acetylcholine (Fig 3A and 3B). With both concentrations of acetylcholine, the vasodilator response was approximately 40% lower in *Sod1-/-* mice fed the control diet than in *Sod1*+/+ mice fed the same diet (P < 0.05). The HM/LF diet resulted in more pronounced decreases in vasodilator response to acetylcholine for all three *Sod1* genotypes (P < 0.001), with the poorest responses detected in *Sod1*-/- mice fed the HM/LF diet (80% decrease vs. *Sod1+/+* fed the control diet). Overall, there was a significant inverse correlation between the dilation response to acetylcholine and plasma tHcy concentration (R = 0.53, P < 0.001; S2 Fig). Collectively, these data demonstrate a combined effect of the hyperhomocysteinemic diet and SOD1 deficiency on endothelial dysfunction that parallels the effects of diet and genotype on vascular superoxide (Fig 2E).

A different pattern of vasomotor responses was observed with two endothelium-independent dilators, nitroprusside and papaverine. Both *Sod1*-/- mice fed the control diet and *Sod1*+/+ mice fed the HM/LF diet exhibited significantly lower responses to the endothelium-independent nitrovasodilator, nitroprusside, compared with *Sod1*+/+ mice fed the control diet (P < 0.05, Fig 3C and 3D). These findings suggested that, like endothelium-dependent vasodilation, endothelium-independent vasodilator responses were adversely affected by both hyperhomocysteinemia and superoxide. Unlike responses to acetylcholine, however, *Sod1*-/- mice did not demonstrate any additional impairment in response to nitroprusside when they were fed the HM/LF diet (Fig 3D). A very similar pattern was seen with papaverine, a direct vascular muscle dilator that is independent of both endothelium and NO. Dilator responses to papaverine were significantly impaired by the HM/LF diet in *Sod1*+/+ and *Sod1*+/- mice but not in *Sod1*-/- mice (P < 0.05, Fig 3E and 3F). These data suggest that SOD1 protects from cerebral vascular dysfunction by limiting the adverse effects of superoxide and/or superoxide-derived ROS on both endothelium and vascular muscle. The more modest effect of SOD1 deficiency on endothelium-independent as compared with endothelium-dependent vasomotor function suggests that the primary impact of superoxide-derived ROS on vasomotor function is to decrease the bioavailability of endothelium-derived NO, with perhaps a secondary influence on smooth muscle and/or other components within the vascular wall.

### Effects of SOD1 deficiency and hyperhomocysteinemia diet on hypertrophy of cerebral arterioles

**Fig 4:**
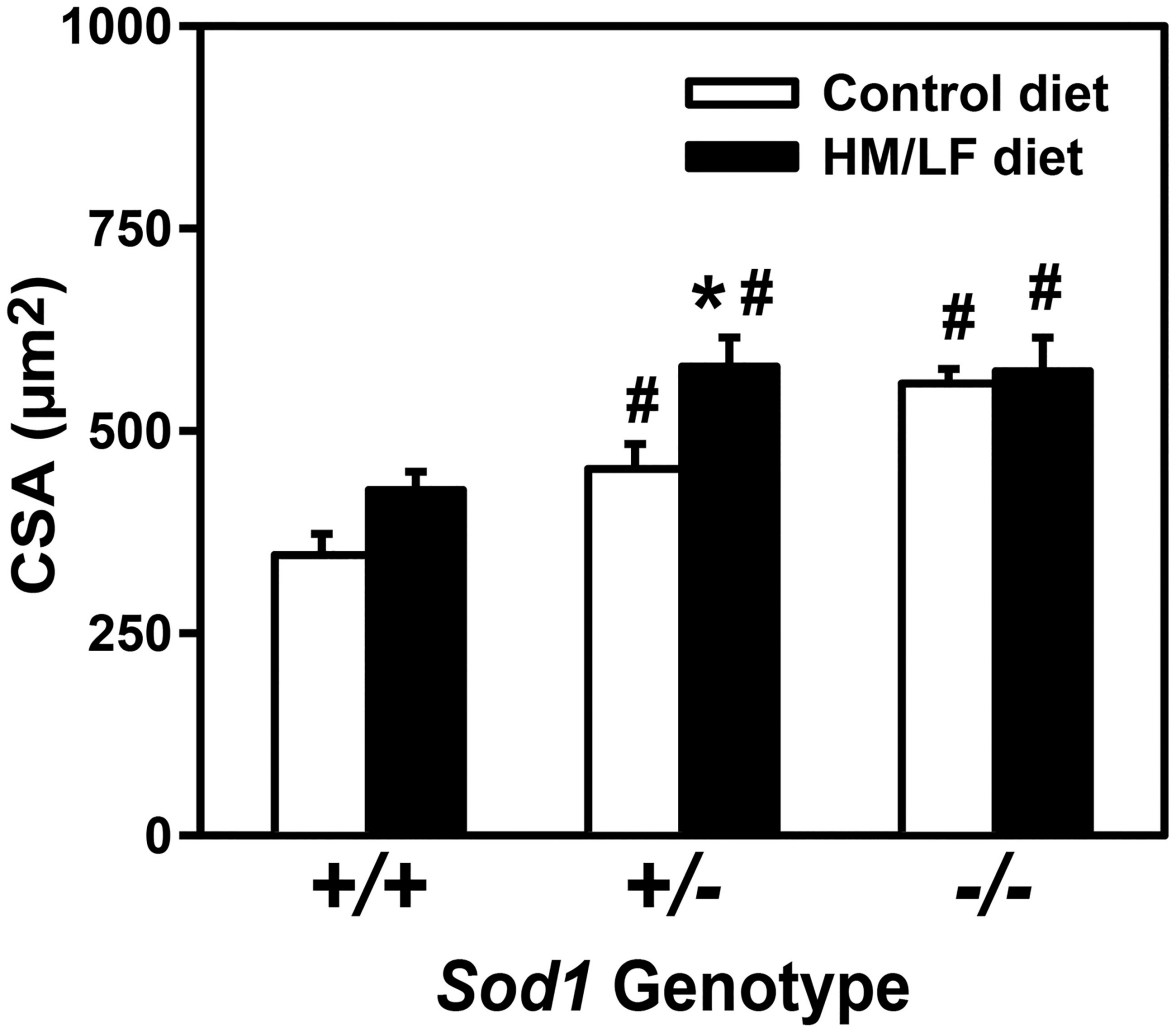
Graded effect of SOD1 deficiency on cerebral arteriolar cross-sectional area is augmented by the HM/LF diet. Total cross-sectional area of vessel wall in cerebral arterioles in *Sod1+/+*, *Sod1+/-*, and *Sod1-/-* mice on either control or HM/LF diet (n = 7–10 mice per group). Values are mean ± SEM. *P < 0.05 compared to control diet; # P<0.05 compared to *Sod1+/+* on the same diet.

To determine whether the hyperhomocysteinemic diet and SOD1 deficiency also perturbed cerebral vascular structure, the cross-sectional area of the cerebral (pial) arteriolar wall was measured after maximal dilation and perfusion fixation. In mice fed the control diet, there was a strong, graded effect of *Sod1* genotype on cross-sectional area, with a 60% increase in area in *Sod1*-/- mice compared with *Sod1*+/+ mice (P < 0.001, Fig 4). The HM/LF diet also had an overall significant effect on cross-sectional area (P < 0.01). Interestingly, the effect of the HM/LF diet on cross-sectional area was highly dependent on the *Sod1* genotype, with the greatest effect of diet observed in *Sod1*+/- mice as demonstrated by a 30% increase with control diet and a 70% increase with HM/LF diet (P < 0.01). The degree of vessel hypertrophy in *Sod1*+/- mice fed the HM/LF diet was equally as severe as that detected in *Sod1*-/- mice fed either diet (Fig 4), suggesting a threshold effect of *Sod1* genotype in the presence of hyperhomocysteinemia.

**Fig 5:**
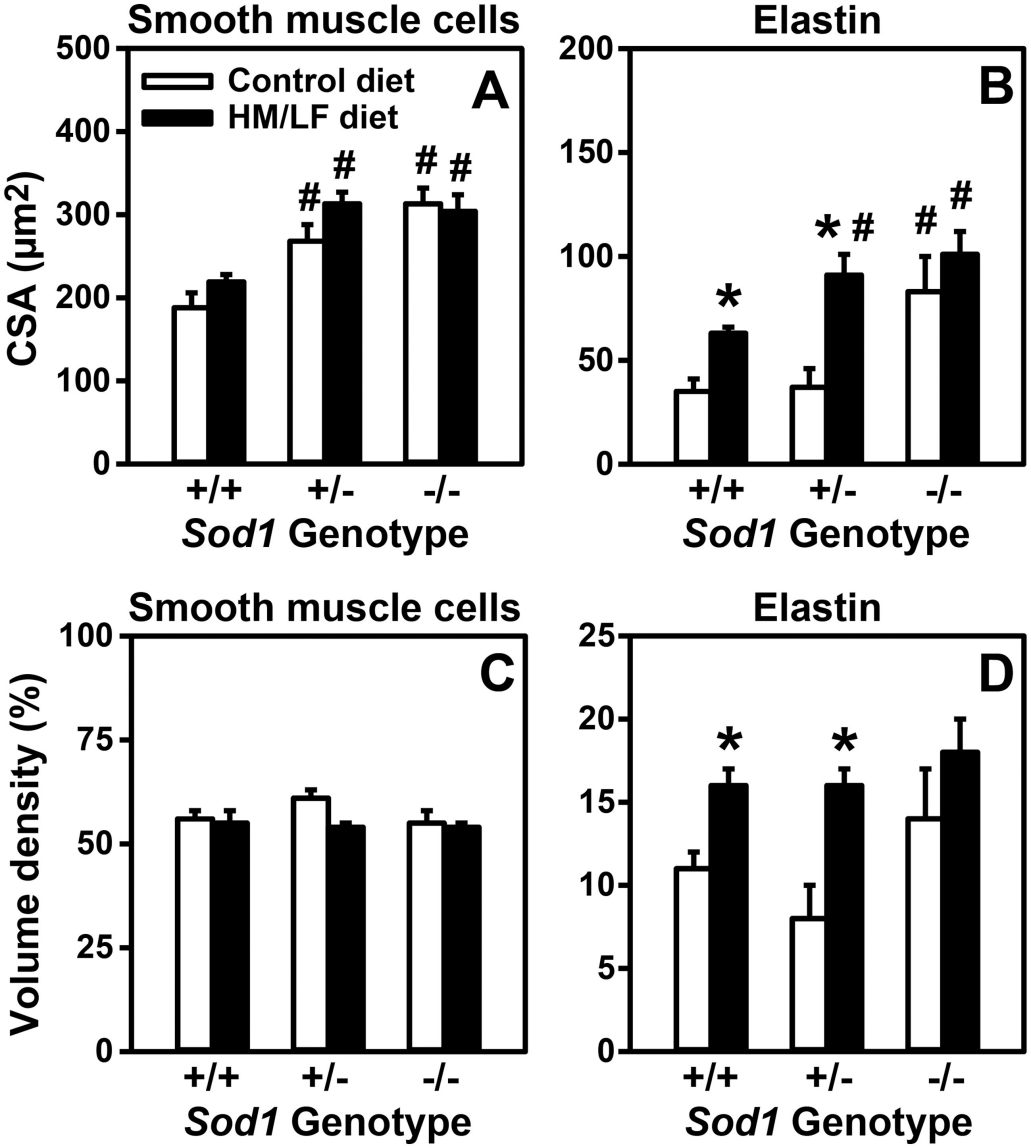
SOD1 deficiency and the HM/LF diet produce differential effects on smooth muscle and elastin components of the vessel wall. Cross-sectional area of smooth muscle (A) and elastin (B) components in cerebral arterioles in *Sod1+/+*, *Sod1+/-*, and *Sod1-/-* mice on either control or HM/LF diet (n = 7–10 mice per group). Percent (%) volume density of smooth muscle (C) and elastin (D) components in cerebral arterioles (n = 7–10 mice per group). Values are mean ± SEM. *P<0.05 compared to control diet; # P < 0.05 compared to *Sod1+/+* on the same diet.

To better understand the structural components of vascular hypertrophy, the cross-sectional area of the major components of the cerebral arteriolar vessel wall were quantitated by electron microscopy (Table 1 and Fig 5). The effects of *Sod1* genotype and the HM/LF diet on the cross-sectional area of smooth muscle (Fig 5A) mirrored the effects of these factors on total cross-sectional area (Fig 4). Deficiency of SOD1 also increased the elastin content as demonstrated by a 2.4-fold higher elastin cross-sectional area in *Sod1*-/- mice compared to *Sod1*+/+ mice fed a control diet (Fig 5B, P<0.05). Interestingly, the HM/LF diet had a particularly strong influence on the content of elastin in the vessel wall. Compared with the control diet, the HM/LF diet increased the cross-sectional area of elastin by 80% in *Sod1*+/+ mice, 150% in *Sod1*+/- mice, and 20% in *Sod1*-/- mice (Table 2, P<0.01). In contrast, the cross-sectional area of collagen, basement membrane, and endothelium were similar regardless of diet or SOD1 deficiency (Table 1).

**Table 1:** Effect of hyperhomocysteinemia on cerebral arteriolar composition in SOD1-deficient mice.

Diet	Control			HM/LF		
*Sod1* Genotype	+/+	+/-	-/-	+/+	+/-	-/-
*N*	6	6	6	5	6	6
*Cross-sectional area (μm*^*2*^*)*						
Smooth muscle	188±18	268±20^#^	334±13^#^	219±9	313±14^#^	304±20^#^
Elastin	35±6	37±9	83±17^#^	63±3*	91±10*^#^	101±11^#^
Collagen	0.8±0.2	1.1±0.7	1.6±0.6	1.1±0.4	1.7±0.7	2.0±0.3
Basement membrane	26±3	33±5	37±4	28±6	44±6	45±8
Endothelium	80±9	100±9	133±13	86±7	134±8^#^	110±3^#^
*Volume density (%)*						
Smooth Muscle	56±2	61±2	58±2	55±3	54±1	54±1
Elastin	11±1	8±2	14±3	16±1*	16±1*	18±2
Collagen	0.3±0.1	0.2±0.1	0.3±0.1	0.3±0.1	0.3±0.1	0.4±0.1
Basement Membrane	8±1	8±1	6±1	7±1	8±1	8±1
Endothelium	25±3	23±2	23±2	22±2	23±2	20±1

Values are mean ± SEM.

*P < 0.05 compared with mice of the same genotype fed the control diet.

^***#***^*P < 0*.*05 compared with* Sod1+/+ *mice fed the same diet*.

**Table 2:** Blood pressures, arterial blood gas parameters, and vascular mechanics.

*Sod1* Genotype	Control Diet			HM/LF Diet		
	+/+	+/-	-/-	+/+	+/-	-/-
*N*	10	12	15	11	10	12
*Prior to deactivation of vascular muscle*						
Systemic arterial mean pressure (mm Hg)						
Unanesthetized	116±3	118±2	110±6	117±4	114±2	106±2^#^
Anesthetized	61±2	59±2	55±2^#^	63±4	59±3	54±2^#^
Cerebral arteriolar pressure (mm Hg)						
Systolic	40±1	39±2	37±2	39±3	41±4	43±2
Diastolic	31±1	29±1	28±2	31±2	31±3	33±2
Mean	34±1	33±1	31±2	34±3	34±3	36±2
Pulse	9±1	10±1	9±1	9±1	10±1	10±1
Arterial blood gases						
pCO_2_	34±2	32±2	32±1	33±2	34±4	34±2
pH	7.26±0.02	7.38±0.02	7.34±0.02	7.26±0.02	7.30±0.03	7.26±0.02
pO_2_	129±10	118±6	120±6	105±3	140±11	128±9
*After deactivation of vascular muscle*						
Cerebral arteriolar diameter (μm)						
Internal	58±3	59±2	64±3	64±3	61±3	67±3
External	61±3	64±2	69±3	68±3	67±3	72±3
E_T_ vs Stress	5.5±0.2	5.2±0.3	4.5±0.2^#^	5.4±0.2	5.2±0.2	4.4±0.3^#^

Measurements prior to deactivation of vascular muscle were obtained at prevailing levels of arterial pressure. Measurements after deactivation of vascular muscle were made at an arteriolar mean pressure of 40 mm Hg. Values of external diameter after deactivation of vascular muscle were calculated from measurements of internal diameter at 40 mm Hg arteriolar pressure and histological measurements of cross-sectional area of the vessel wall. E_T_ vs Stress: slope of tangential elastic modulus (E_T_) versus stress. Values are mean ± SEM.

^**#**^P < 0.05 compared with *Sod1+/+* mice fed the same diet.

We next evaluated the impact of SOD1 deficiency and diet on the relative proportions of smooth muscle and elastin within the vessel wall. The largest constituent of the vessel wall was the smooth muscle component (Table 1). The ratio of smooth muscle to the total size of the vessel (volume density) was not altered by either the HM/LF diet or SOD1 deficiency, as evidenced by a similar percent volume density among all groups (Fig 5C, Table 1). SOD1 deficiency alone did not significantly alter the elastin percent volume density, but a significant increase in elastin volume density was observed in *Sod1*+/+ and *Sod1*+/- mice fed the HM/LF diet (P < 0.05, Fig 5D).

### Blood pressure and vascular mechanics

To understand whether vascular remodeling contributes to the diet- and SOD1 deficiency-induced changes in vascular structure, we measured blood pressure and vascular mechanics of cerebral arterioles. As expected [22], *Sod1*-/- mice had lower systemic arterial mean pressures than *Sod1*+/+ mice in both the unanesthetized and anesthetized states (P<0.05) (Table 2). The hyperhomocysteinemia diet did not influence systemic arterial mean blood pressure (Table 2). Cerebral arteriolar pressures under anesthesia did not differ significantly among any groups of mice. Next, we measured the internal and external elastic diameters of cerebral arterioles after maximal dilation induced by deactivation of vascular muscle with EDTA (Table 2) as a measure of remodeling. No significant differences in internal or external diameter were found between groups regardless of *Sod1* genotype or diet (Table 2). The absence of a reduction in internal or external diameter with SOD1 deficiency and/or the HM/LF diet indicates that the structural changes cannot be attributed to inward remodeling. Finally, the slope of tangential elastic modulus (Eт) versus stress, a measure of vessel rigidity, was significantly decreased in *Sod1-*/- mice compared with *Sod1+*/+ mice fed either diet (Table 2), consistent with the increase in elastin content observed in SOD1-deficient vessels.

## Discussion

In this study, we utilized SOD1-deficient mice to determine the pathophysiological importance of superoxide-derived ROS in the cerebral vascular consequences of diet-induced hyperhomocysteinemia. As reported previously [19], we found that mice fed the hyperhomocysteinemic HM/LF diet exhibited increased vascular superoxide levels and marked endothelial vasomotor dysfunction in cerebral blood vessels. The novel findings of the current study are: 1) The effects of the hyperhomocysteinemic diet on superoxide levels and endothelial vasomotor dysfunction were exacerbated in mice with SOD1 deficiency; 2) We also observed modestly impaired vasomotor responses to the endothelium-independent responses nitroprusside or papaverine in mice with hyperhomocysteinemia or deficiency of SOD1; 3) Finally, we found that endogenous SOD1 protects against cerebral vascular hypertrophy, and parallel increases in the content of smooth muscle and elastin in the cerebral arteriolar wall, in mice with hyperhomocysteinemia. Taken together, these findings implicate superoxide and/or superoxide-derived ROS as key redox mediators of abnormal cerebral vascular structure and function in hyperhomocysteinemia.

We chose to target SOD1 in this study because it is the most abundant cellular isoform of SOD in vascular tissues [20], it is a direct regulator of superoxide, and superoxide-derived ROS have been proposed as mediators of vascular dysfunction and vascular structural abnormalities in hyperhomocysteinemia [16,17]. As expected, we found that mice fed the HM/LF diet had mild/moderate hyperhomocysteinemia. The hyperhomocysteinemic response to the HM/LF diet was not altered by deficiency of SOD1, which suggests that dysregulation of superoxide is a consequence, rather than a cause of hyperhomocysteinemia. It remains possible that some of the observed effects of the HM/LF diet could be due to altered expression of other antioxidant enzymes during hyperhomocysteinemia. However, this is unlikely in our model because we have demonstrated previously that diet-induced hyperhomocysteinemia in mice does not influence tissue levels of catalase, glutathione peroxidase, or other isoforms of SOD [25].

SOD1 appears to protect from the adverse vascular effects of superoxide on both endothelium and vascular muscle. These protective mechanisms may be inter-related, since endothelium-derived NO can scavenge superoxide and blunt hypertrophy of vascular muscle [31] and superoxide may cause decreased vascular responses to both endothelium-derived NO and exogenous NO donors such as nitroprusside [32]. In contrast, papaverine is thought to produce relaxation of smooth muscle via direct effects on voltage-dependent calcium channels [28], independently of endothelium, NO, or guanylate cyclase [27]. Therefore, additional work is needed to define the mechanisms that account for the observed effect of the HM/LF diet on papaverine-induced vasodilation. Interestingly, the effect of SOD1 deficiency on endothelium-dependent vasomotor function was much more robust that its effects on endothelium-independent vasodilatation, which suggests that the primary impact of superoxide-derived ROS is to decrease the bioavailability of endothelium-derived NO, with perhaps a secondary influence on smooth muscle and/or other components within the vascular wall.

The clear additive effects of SOD1 deficiency and the hyperhomocysteinemic HM/LF diet on endothelial dysfunction suggests that, in addition to superoxide, other factors also may contribute to impaired endothelium-dependent vasodilatation. One possible mediator is H_2_O_2_, given that: 1) expression of the NADPH oxidase catalytic subunit Nox4, which can generate H_2_O_2_ even in the absence of SOD [33], is elevated in the aorta of mice with hyperhomocysteinemia [30]; 2) diet-induced hyperhomocysteineima is associated with increased H_2_O_2_ in cerebral arterioles [17]; and 3) it has been demonstrated that glutathione peroxidase-1, which catalytically eliminates H_2_O_2_, protects non-cerebral vessels from endothelial dysfunction in mice with diet-induced hyperhomocysteinemia [25,34]. Another possible mechanism of endothelial dysfunction in mice fed the HM/LF diet is that low dietary folate may cause depletion of reduced tetrahydrobiopertin, leading to decreased eNOS activity [35]. Thus, while our data implicate superoxide as a primary mediator of the cerebral vascular effects of diet-induced hyperhomocysteinemia, other factors in addition to superoxide may play a contributing role.

We also found that the hyperhomocysteinemic diet had a major influence on superoxide-related structural changes in cerebral arterioles. Consistent with previous findings [29], we observed cerebral vascular hypertrophy (increased cross-sectional area of pial arterioles) with SOD1 deficiency, with a clear *Sod1* gene dose dependence in *Sod1+/- and Sod1-/-* mice (Fig 4). Moreover, the hyperhomocysteinemic diet appeared to sensitize to the hypertrophic phenotype of SOD1-deficient mice, best illustrated by the markedly increased severity of hypertrophy in heterozygous *Sod1*+/- mice fed the HM/LF diet. We interpret these findings as suggestive of a threshold effect in which maximal increases in cross-sectional area were produced by either the HM/LF diet in *Sod1+/-* mice or by homozygous Sod1 deficiency in Sod1-/- mice fed the control diet. Adding the HM/LF diet to *Sod1-/-* mice did not produce any further increase in cross-sectional area, likely because the hypertrophic effect was already maximized by complete SOD1 deficiency. In contrast, a partial deficiency of SOD1 in *Sod1+/-* mice was sufficient to drive the phenotype only in the setting of diet-induced hyperhomocysteinemia.

IThe vascular hypertrophy was not accompanied by changes in internal cerebral arteriolar, inward remodeling, or hypertension. Characterization of the vessel wall components revealed that the major contributor to vascular hypertrophy was a proportional increase in smooth muscle, which is consistent with previous reports that homocysteine induces smooth muscle proliferation *in vitro* [36–38]. In addition to effects on the smooth muscle cell component of the vascular wall, the elastin content was disproportionately increased in response to the hyperhomocysteinemic diet. Alterations in elastin content and structure have been reported previously in several different animal models of hyperhomocysteinemia [39,40].

## Conclusions

The data reported herein implicate superoxide as a key mediator of cerebral vascular dysfunction and vascular hypertrophy in diet-induced hyperhomocysteinemia. These findings may help provide a mechanistic underpinning for clinical observations implicating mild hyperhomocysteinemia as an independent risk factor for cerebral vascular disease and ischemic stroke. In addition to homocysteine-lowering therapies such as folic acid supplementation, future efforts to prevent the cerebral vascular complications of hyperhomocysteinemia might target superoxide-dependent mechanisms.

## Supporting information

S1 Fig Timeline of experimental design. At 4 weeks of age, *Sod1*+/+, *Sod1*+/-, and *Sod1*-/- mice were randomized (R) to the high methionine/low folate (HM/LF) diet or the control diet. After 5–10 months (i.e., at 6–11 months of age) the indicated experimental assessments were performed.
(PDF)

S2 Fig Correlation between plasma tHcy level and dilatation of cerebral arterioles to acetylcholine. Circles, *Sod1*+/+ mice; squares, *Sod1*+/- mice; triangles, *Sod1*-/- mice; open symbols, control diet; filled symbols, HM/LF diet. N = 7–10 mice per group.
(PDF)
