# Supplementary material for: Deficiency of superoxide dismutase promotes cerebral vascular hypertrophy and vascular dysfunction in hyperhomocysteinemia
Source: PLoS One. 2017 Apr 17;12(4):e0175732. doi: 10.1371/journal.pone.0175732 (PMC5393600; doi:10.1371/journal.pone.0175732)
Supplement: S1 Fig — At 4 weeks of age, Sod1+/+, Sod1+/-, and Sod1-/- mice were randomized (R) to the high methionine/low folate (HM/LF) diet or the control diet. After 5–10 months (i.e., at 6–11 months of age) the indicated experimental assessments were performed. (PDF) [file pone.0175732.s001.pdf]

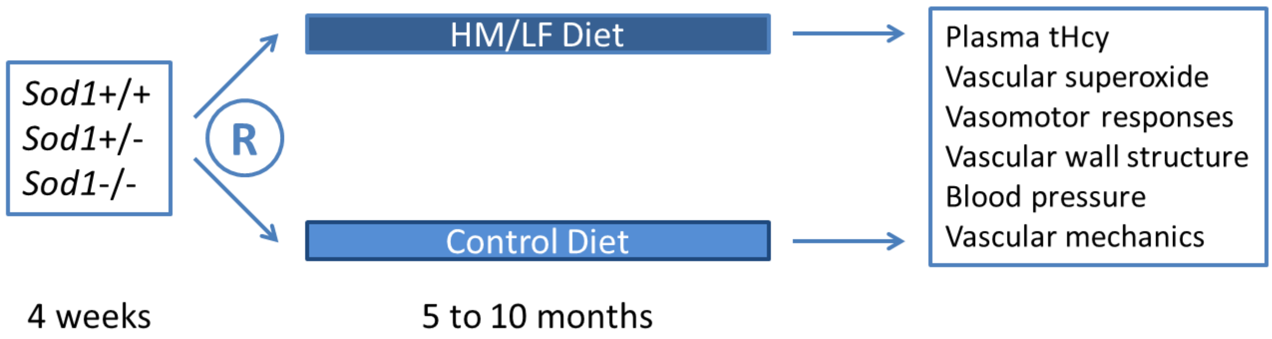

S1 Fig. Timeline of experimental design. At 4 weeks of age, *Sod1*+/+, *Sod1*+/-, and *Sod1*-/- mice were randomized (R) to the high methionine/low folate (HM/LF) diet or the control diet. After 5-10 months (i.e., at 6-11 months of age) the indicated experimental assessments were performed.
