# Supplementary material for: Deficiency of superoxide dismutase promotes cerebral vascular hypertrophy and vascular dysfunction in hyperhomocysteinemia
Source: PLoS One. 2017 Apr 17;12(4):e0175732. doi: 10.1371/journal.pone.0175732 (PMC5393600; doi:10.1371/journal.pone.0175732)
Supplement: S2 Fig — Circles, Sod1+/+ mice; squares, Sod1+/- mice; triangles, Sod1-/- mice; open symbols, control diet; filled symbols, HM/LF diet. N = 7–10 mice per group. (PDF) [file pone.0175732.s002.pdf]

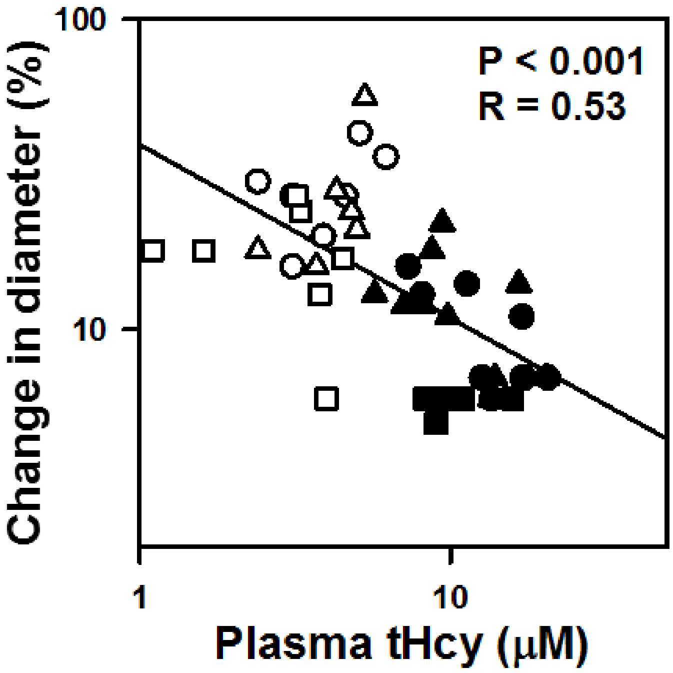

S2 Fig. Correlation between plasma tHcy level and dilatation of cerebral arterioles to acetylcholine. Circles, *Sod1*+/+ mice; squares, *Sod1*+/- mice; triangles, *Sod1*-/- mice; open symbols, control diet; filled symbols, HM/LF diet. N=7-10 mice per group.
